# *N*′-(3,5-Dibromo-2-hy­droxy­benzyl­idene)-4-nitro­benzohydrazide methanol monosolvate

**DOI:** 10.1107/S1600536811031187

**Published:** 2011-08-11

**Authors:** Xin Zhou, Shu-Tao Gao, Jing-Jun Ma

**Affiliations:** a Hebei Key Laboratory of Bioinorganic Chemistry, College of Sciences, Agricultural University of Hebei, Baoding 071001, People’s Republic of China

## Abstract

The title compound, C_14_H_9_Br_2_N_3_O_4_·CH_4_O, was obtained as the product of the reaction of 3,5-dibromo­salicyl­aldehyde with 4-nitro­benzohydrazide in methanol. The benzohydrazide mol­ecule is nearly planar, with a maximum deviation of 0.126 (2) Å. The mean planes of the two benzene rings make a dihedral angle of 9.3 (3)°. Intra­molecular O—H⋯N and O—H⋯Br inter­actions are observed in the benzohydrazide mol­ecule. In the crystal, pairs of adjacent benzohydrazide mol­ecules are linked by two methanol mol­ecules through inter­molecular O—H⋯O and N—H⋯O hydrogen bonds, forming a dimer.

## Related literature

For the biological activity of benzohydrazide compounds, see: El-Sayed *et al.* (2011 ▶); Horiuchi *et al.* (2009 ▶). For coordination compounds involving benzohydrazides, see: El-Dissouky *et al.* (2010 ▶); Zhang *et al.* (2010 ▶). For standard bond lengths, see: Allen *et al.* (1987 ▶). For related structures, see: Suleiman Gwaram *et al.* (2010 ▶); Dai & Mao (2010 ▶); Ban (2010 ▶).
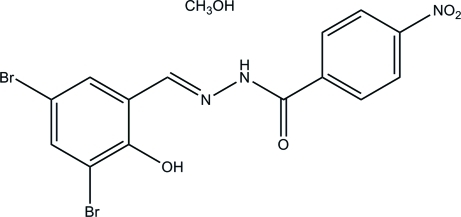


## Experimental

### 

#### Crystal data

C_14_H_9_Br_2_N_3_O_4_·CH_4_O
*M* *_r_* = 475.10
Monoclinic, 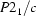

*a* = 7.576 (2) Å
*b* = 13.602 (2) Å
*c* = 17.230 (3) Å
β = 106.816 (3)°
*V* = 1699.6 (6) Å^3^
*Z* = 4
Mo *K*α radiation
μ = 4.80 mm^−1^
*T* = 298 K
0.30 × 0.27 × 0.27 mm

#### Data collection

Bruker SMART 1K CCD area-detector diffractometer
Absorption correction: multi-scan (*SADABS*; Sheldrick, 2004 ▶) *T*_min_ = 0.327, *T*_max_ = 0.357
10168 measured reflections
3685 independent reflections
2011 reflections with *I* > 2σ(*I*)
*R*_int_ = 0.075

#### Refinement

*R*[*F* ^2^ > 2σ(*F* ^2^)] = 0.049
*wR*(*F* ^2^) = 0.103
*S* = 1.00
3685 reflections
227 parameters
H-atom parameters constrained
Δρ_max_ = 0.53 e Å^−3^
Δρ_min_ = −0.52 e Å^−3^

### 

Data collection: *SMART* (Bruker, 2001 ▶); cell refinement: *SAINT* (Bruker, 2001 ▶); data reduction: *SAINT*; program(s) used to solve structure: *SHELXTL* (Sheldrick, 2008 ▶); program(s) used to refine structure: *SHELXTL*; molecular graphics: *SHELXTL*; software used to prepare material for publication: *SHELXTL*.

## Supplementary Material

Crystal structure: contains datablock(s) I, global. DOI: 10.1107/S1600536811031187/qm2019sup1.cif

Structure factors: contains datablock(s) I. DOI: 10.1107/S1600536811031187/qm2019Isup2.hkl

Supplementary material file. DOI: 10.1107/S1600536811031187/qm2019Isup3.cml

Additional supplementary materials:  crystallographic information; 3D view; checkCIF report

## Figures and Tables

**Table 1:** Hydrogen-bond geometry (Å, °)

*D*—H⋯*A*	*D*—H	H⋯*A*	*D*⋯*A*	*D*—H⋯*A*
O1—H1⋯N1	0.82	1.94	2.642 (5)	143
N2—H2⋯O5^i^	0.90	1.91	2.800 (5)	169
O5—H5⋯O2^ii^	0.82	2.24	2.991 (6)	153
O5—H5⋯Br2	0.82	3.09	3.708 (4)	134

Symmetry codes: (i) 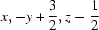; (ii) 
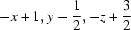
.

**Fig. 1.**
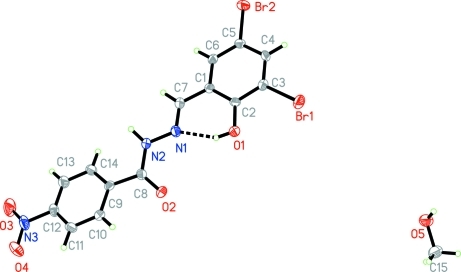
The molecular structure of the title compound, with displacement ellipsoids drawn at the 30% probability level.

**Fig. 2.**
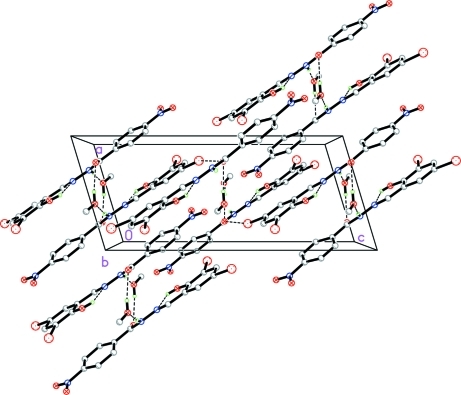
The packing of the title compound. Hydrogen bonds are shown as dashed lines. H atoms not related to the hydrogen bonding are omitted for clarity.

## supplementary crystallographic information

### Comment

Benzohydrazide compounds are well known for their biological activities (El-Sayed *et al.*, 2011; Horiuchi *et al.*, 2009). In addition, benzohydrazide compounds have also been used as versatile ligands in coordination chemistry (El-Dissouky *et al.*, 2010). As a contribution to a structural study on hydrazone compounds, we present here the crystal structure of the title compound.

The compound contains a benzohydrazide molecule and a methanol molecule, as shown in Fig. 1. The bond distances and angles are within normal ranges (Allen *et al.*, 1987) and are agree well with the corresponding bond distances and angles reported in closely related compounds (Suleiman Gwaram *et al.*, 2010; Dai & Mao, 2010; Ban, 2010). The benzohydrazide molecule is nearly planar, with a maximum deviation of 0.126 (2) Å. The mean planes of the two benzene rings make a dihedral angle of 9.3 (3)°.

In the crystal structure of the title compound, the adjacent two benzohydrazide molecules are linked by two methanol molecules through intermolecular O—H···O and N—H···O hydrogen bonds, to form a dimer (Table 1, Fig. 2).

### Experimental

To a methanol solution (20 ml) of 3,5-dibromosalicylaldehyde (0.1 mmol, 28.0 mg) and 4-nitrobenzohydrazide (0.1 mmol, 18.1 mg), a few drops of acetic acid were added. The mixture was refluxed for 1 h and then cooled to room temperature. The white crystalline solid was collected by filtration, washed with cold methanol and dried in air. Single crystals suitable for X-ray diffraction were obtained by slow evaporation of a methanol solution of the product in air.

### Refinement

H2 atoms bonded to N2 was located in a difference map and refined with distance restraint to 0.90 (1) Å. Other H atoms were positioned geometrically and refined using a riding model, with C—H = 0.93–0.96 Å and with *U*_iso_(H) = 1.2*U*_eq_(C) and 1.5*U*_eq_(O and C15).

### Crystal data

**Table:**

C_14_H_9_Br_2_N_3_O_4_·CH_4_O	*F*(000) = 936
*M**_r_* = 475.10	*D*_x_ = 1.857 Mg m^−^^3^
Monoclinic, *P*2_1_/*c*	Mo *K*α radiation, λ = 0.71073 Å
*a* = 7.576 (2) Å	Cell parameters from 1293 reflections
*b* = 13.602 (2) Å	θ = 2.5–24.5°
*c* = 17.230 (3) Å	µ = 4.80 mm^−^^1^
β = 106.816 (3)°	*T* = 298 K
*V* = 1699.6 (6) Å^3^	Block, yellow
*Z* = 4	0.30 × 0.27 × 0.27 mm

### Data collection

**Table:**

Bruker SMART 1K CCD area-detector diffractometer	3685 independent reflections
Radiation source: fine-focus sealed tube	2011 reflections with *I* > 2σ(*I*)
graphite	*R*_int_ = 0.075
ω scans	θ_max_ = 27.0°, θ_min_ = 2.5°
Absorption correction: multi-scan (*SADABS*; Sheldrick, 2004)	*h* = −9→9
*T*_min_ = 0.327, *T*_max_ = 0.357	*k* = −15→17
10168 measured reflections	*l* = −22→13

### Refinement

**Table:**

Refinement on *F*^2^	Primary atom site location: structure-invariant direct methods
Least-squares matrix: full	Secondary atom site location: difference Fourier map
*R*[*F*^2^ > 2σ(*F*^2^)] = 0.049	Hydrogen site location: inferred from neighbouring sites
*wR*(*F*^2^) = 0.103	H-atom parameters constrained
*S* = 1.00	*w* = 1/[σ^2^(*F*_o_^2^) + (0.*P*)^2^] where *P* = (*F*_o_^2^ + 2*F*_c_^2^)/3
3685 reflections	(Δ/σ)_max_ = 0.001
227 parameters	Δρ_max_ = 0.53 e Å^−^^3^
0 restraints	Δρ_min_ = −0.52 e Å^−^^3^

### Special details

**Table:**

Geometry. All e.s.d.'s (except the e.s.d. in the dihedral angle between two l.s. planes) are estimated using the full covariance matrix. The cell e.s.d.'s are taken into account individually in the estimation of e.s.d.'s in distances, angles and torsion angles; correlations between e.s.d.'s in cell parameters are only used when they are defined by crystal symmetry. An approximate (isotropic) treatment of cell e.s.d.'s is used for estimating e.s.d.'s involving l.s. planes.
Refinement. Refinement of *F*^2^ against ALL reflections. The weighted *R*-factor *wR* and goodness of fit *S* are based on *F*^2^, conventional *R*-factors *R* are based on *F*, with *F* set to zero for negative *F*^2^. The threshold expression of *F*^2^ > σ(*F*^2^) is used only for calculating *R*-factors(gt) *etc*. and is not relevant to the choice of reflections for refinement. *R*-factors based on *F*^2^ are statistically about twice as large as those based on *F*, and *R*- factors based on ALL data will be even larger.

### Fractional atomic coordinates and isotropic or equivalent isotropic displacement parameters (Å^2^)

**Table:**

	*x*	*y*	*z*	*U*_iso_*/*U*_eq_	
Br1	0.83099 (8)	1.22176 (4)	0.84550 (4)	0.0533 (2)	
Br2	0.79926 (8)	0.82702 (4)	0.94026 (3)	0.0492 (2)	
O1	0.5709 (5)	1.1483 (2)	0.6907 (2)	0.0470 (10)	
H1	0.5200	1.1265	0.6454	0.056*	
O2	0.2149 (5)	1.1321 (3)	0.4660 (2)	0.0584 (11)	
O3	−0.3295 (6)	0.8090 (4)	0.1561 (3)	0.0822 (16)	
O4	−0.3194 (7)	0.9500 (4)	0.1026 (3)	0.0953 (17)	
N1	0.3873 (5)	1.0128 (3)	0.5892 (2)	0.0384 (11)	
N2	0.2818 (5)	0.9780 (3)	0.5145 (2)	0.0404 (11)	
H2	0.3046	0.9146	0.5048	0.048*	
N3	−0.2750 (6)	0.8932 (5)	0.1592 (3)	0.0576 (15)	
C1	0.5601 (6)	0.9761 (3)	0.7247 (3)	0.0320 (12)	
C2	0.6184 (6)	1.0736 (4)	0.7435 (3)	0.0321 (12)	
C3	0.7351 (6)	1.0935 (4)	0.8209 (3)	0.0355 (13)	
C4	0.7889 (6)	1.0222 (4)	0.8791 (3)	0.0383 (13)	
H4	0.8662	1.0374	0.9303	0.046*	
C5	0.7259 (6)	0.9266 (4)	0.8603 (3)	0.0355 (12)	
C6	0.6146 (6)	0.9041 (3)	0.7845 (3)	0.0347 (12)	
H6	0.5747	0.8397	0.7727	0.042*	
C7	0.4506 (7)	0.9483 (4)	0.6443 (3)	0.0388 (13)	
H7	0.4256	0.8821	0.6323	0.047*	
C8	0.1984 (7)	1.0439 (4)	0.4568 (3)	0.0365 (13)	
C9	0.0789 (6)	0.9994 (4)	0.3795 (3)	0.0326 (12)	
C10	0.0309 (7)	1.0598 (4)	0.3111 (3)	0.0397 (13)	
H10	0.0768	1.1235	0.3141	0.048*	
C11	−0.0849 (7)	1.0247 (4)	0.2391 (3)	0.0429 (14)	
H11	−0.1175	1.0645	0.1932	0.052*	
C12	−0.1505 (7)	0.9317 (4)	0.2361 (3)	0.0417 (14)	
C13	−0.1068 (7)	0.8708 (4)	0.3030 (3)	0.0498 (15)	
H13	−0.1535	0.8072	0.2993	0.060*	
C14	0.0068 (7)	0.9054 (4)	0.3751 (3)	0.0419 (14)	
H14	0.0352	0.8656	0.4209	0.050*	
O5	0.4000 (5)	0.7107 (3)	0.9877 (2)	0.0612 (11)	
H5	0.5128	0.7097	0.9988	0.073*	
C15	0.3336 (8)	0.8036 (4)	0.9540 (4)	0.0627 (18)	
H15A	0.3740	0.8154	0.9069	0.094*	
H15B	0.3807	0.8542	0.9934	0.094*	
H15C	0.2013	0.8039	0.9389	0.094*	

### Atomic displacement parameters (Å^2^)

**Table:**

	*U*^11^	*U*^22^	*U*^33^	*U*^12^	*U*^13^	*U*^23^
Br1	0.0592 (4)	0.0348 (4)	0.0627 (4)	−0.0076 (3)	0.0125 (3)	−0.0113 (3)
Br2	0.0522 (4)	0.0489 (4)	0.0435 (4)	0.0081 (3)	0.0087 (3)	0.0129 (3)
O1	0.064 (2)	0.031 (2)	0.038 (2)	0.0028 (18)	0.0037 (19)	0.0001 (17)
O2	0.086 (3)	0.027 (2)	0.050 (3)	0.004 (2)	−0.001 (2)	−0.0070 (19)
O3	0.072 (3)	0.088 (4)	0.073 (3)	−0.020 (3)	0.000 (3)	−0.039 (3)
O4	0.111 (4)	0.104 (4)	0.042 (3)	0.017 (3)	−0.023 (3)	−0.004 (3)
N1	0.035 (2)	0.043 (3)	0.034 (3)	0.001 (2)	0.006 (2)	−0.008 (2)
N2	0.051 (3)	0.033 (3)	0.033 (3)	0.004 (2)	0.006 (2)	−0.007 (2)
N3	0.042 (3)	0.086 (5)	0.037 (3)	0.010 (3)	−0.001 (3)	−0.022 (3)
C1	0.032 (3)	0.028 (3)	0.034 (3)	−0.001 (2)	0.007 (2)	−0.001 (2)
C2	0.032 (3)	0.034 (3)	0.029 (3)	0.008 (2)	0.008 (2)	0.005 (2)
C3	0.033 (3)	0.032 (3)	0.042 (3)	−0.002 (2)	0.013 (3)	−0.007 (2)
C4	0.032 (3)	0.044 (4)	0.032 (3)	0.003 (2)	−0.001 (2)	−0.010 (3)
C5	0.035 (3)	0.033 (3)	0.037 (3)	0.003 (2)	0.008 (3)	0.005 (2)
C6	0.040 (3)	0.026 (3)	0.037 (3)	−0.001 (2)	0.011 (3)	−0.002 (2)
C7	0.045 (3)	0.035 (3)	0.035 (3)	−0.001 (3)	0.010 (3)	−0.005 (3)
C8	0.036 (3)	0.039 (4)	0.034 (3)	0.002 (3)	0.009 (3)	−0.003 (3)
C9	0.035 (3)	0.029 (3)	0.035 (3)	0.007 (2)	0.013 (3)	0.000 (2)
C10	0.044 (3)	0.035 (3)	0.039 (3)	0.008 (3)	0.010 (3)	−0.002 (3)
C11	0.045 (3)	0.053 (4)	0.031 (3)	0.015 (3)	0.011 (3)	0.009 (3)
C12	0.029 (3)	0.057 (4)	0.037 (3)	0.000 (3)	0.006 (3)	−0.012 (3)
C13	0.058 (4)	0.043 (4)	0.043 (4)	−0.006 (3)	0.006 (3)	−0.004 (3)
C14	0.045 (3)	0.037 (3)	0.038 (3)	−0.004 (3)	0.004 (3)	0.003 (3)
O5	0.050 (2)	0.056 (3)	0.070 (3)	−0.007 (2)	0.004 (2)	0.014 (2)
C15	0.058 (4)	0.048 (4)	0.079 (5)	0.002 (3)	0.015 (4)	0.007 (3)

### Geometric parameters (Å, °)

**Table:**

Br1—C3	1.890 (5)	C5—C6	1.368 (6)
Br2—C5	1.898 (5)	C6—H6	0.9300
O1—C2	1.341 (5)	C7—H7	0.9300
O1—H1	0.8200	C8—C9	1.505 (6)
O2—C8	1.212 (5)	C9—C14	1.383 (6)
O3—N3	1.214 (6)	C9—C10	1.396 (6)
O4—N3	1.213 (6)	C10—C11	1.381 (7)
N1—C7	1.280 (6)	C10—H10	0.9300
N1—N2	1.389 (5)	C11—C12	1.355 (7)
N2—C8	1.352 (6)	C11—H11	0.9300
N2—H2	0.9035	C12—C13	1.379 (7)
N3—C12	1.483 (6)	C13—C14	1.374 (7)
C1—C6	1.395 (6)	C13—H13	0.9300
C1—C2	1.406 (6)	C14—H14	0.9300
C1—C7	1.444 (6)	O5—C15	1.420 (6)
C2—C3	1.398 (6)	O5—H5	0.8200
C3—C4	1.369 (6)	C15—H15A	0.9600
C4—C5	1.390 (6)	C15—H15B	0.9600
C4—H4	0.9300	C15—H15C	0.9600
			
C2—O1—H1	109.4	O2—C8—N2	123.5 (5)
C7—N1—N2	116.4 (4)	O2—C8—C9	121.8 (5)
C8—N2—N1	118.5 (4)	N2—C8—C9	114.7 (5)
C8—N2—H2	124.8	C14—C9—C10	119.7 (5)
N1—N2—H2	114.0	C14—C9—C8	123.2 (5)
O4—N3—O3	123.7 (5)	C10—C9—C8	117.0 (5)
O4—N3—C12	116.8 (6)	C11—C10—C9	119.8 (5)
O3—N3—C12	119.5 (6)	C11—C10—H10	120.1
C6—C1—C2	119.1 (4)	C9—C10—H10	120.1
C6—C1—C7	119.3 (5)	C12—C11—C10	119.2 (5)
C2—C1—C7	121.5 (4)	C12—C11—H11	120.4
O1—C2—C3	118.2 (4)	C10—C11—H11	120.4
O1—C2—C1	123.6 (4)	C11—C12—C13	122.2 (5)
C3—C2—C1	118.2 (4)	C11—C12—N3	119.6 (5)
C4—C3—C2	122.2 (5)	C13—C12—N3	118.2 (5)
C4—C3—Br1	118.4 (4)	C14—C13—C12	119.1 (5)
C2—C3—Br1	119.3 (4)	C14—C13—H13	120.5
C3—C4—C5	118.9 (4)	C12—C13—H13	120.5
C3—C4—H4	120.6	C13—C14—C9	120.0 (5)
C5—C4—H4	120.6	C13—C14—H14	120.0
C6—C5—C4	120.5 (4)	C9—C14—H14	120.0
C6—C5—Br2	120.2 (4)	C15—O5—H5	109.4
C4—C5—Br2	119.3 (4)	O5—C15—H15A	109.5
C5—C6—C1	121.1 (5)	O5—C15—H15B	109.5
C5—C6—H6	119.4	H15A—C15—H15B	109.5
C1—C6—H6	119.4	O5—C15—H15C	109.5
N1—C7—C1	121.3 (5)	H15A—C15—H15C	109.5
N1—C7—H7	119.4	H15B—C15—H15C	109.5
C1—C7—H7	119.4		

### Hydrogen-bond geometry (Å, °)

**Table:**

*D*—H···*A*	*D*—H	H···*A*	*D*···*A*	*D*—H···*A*
O1—H1···N1	0.82	1.94	2.642 (5)	143.
N2—H2···O5^i^	0.90	1.91	2.800 (5)	169.
O5—H5···O2^ii^	0.82	2.24	2.991 (6)	153.
O5—H5···Br2	0.82	3.09	3.708 (4)	134.

Symmetry codes: (i) *x*, −*y*+3/2, *z*−1/2; (ii) −*x*+1, *y*−1/2, −*z*+3/2.
